# The Relationship of Housing and Population Health: A 30-Year Retrospective Analysis

**DOI:** 10.1289/ehp.0800086

**Published:** 2008-12-16

**Authors:** David E. Jacobs, Jonathan Wilson, Sherry L. Dixon, Janet Smith, Anne Evens

**Affiliations:** 1 National Center for Healthy Housing, Columbia, Maryland, USA;; 2 University of Illinois at Chicago, Chicago, Illinois, USA

**Keywords:** American Housing Survey (AHS), asthma, health disparities, healthy housing, housing, lead poisoning, National Health and Nutrition Examination Survey (NHANES)

## Abstract

**Objective:**

We analyzed the relationship between health status and housing quality over time.

**Methods:**

We combined data from two nationally representative longitudinal surveys of the U.S. population and its housing, the National Health and Nutrition Examination Survey and the American Housing Survey, respectively. We identified housing and health trends from approximately 1970 to 2000, after excluding those trends for which data were missing or where we found no plausible association or change in trend.

**Results:**

Changes in housing include construction type, proportion of rental versus home ownership, age, density, size, moisture, pests, broken windows, ventilation and air conditioning, and water leaks. Changes in health measures include asthma, respiratory illness, obesity and diabetes, and lead poisoning, among others. The results suggest ecologic trends in childhood lead poisoning follow housing age, water leaks, and ventilation; asthma follows ventilation, windows, and age; overweight trends follow ventilation; blood pressure trends follow community measures; and health disparities have not changed greatly.

**Conclusions:**

Housing trends are consistent with certain health trends over time. Future national longitudinal surveys should include health, housing, and community metrics within a single integrated design, instead of separate surveys, in order to develop reliable indicators of how housing changes affect population health and how to best target resources. Little progress has been made in reducing the health and housing disparities of disadvantaged groups, with the notable exception of childhood lead poisoning caused by exposure to lead-based paint hazards. Use of these and other data sets to create reliable integrated indicators of health and housing quality are needed.

Physical changes to the nation’s housing stock are a continuous process responding to consumer demand, technological innovation, and periodic policy interventions (Galster 2008). Since World War II, most changes to the home environment in the United States have aimed to improve durability, energy conservation, general comfort, and security, as well as aesthetics. However, few have been intended to improve health directly. Although some housing changes produce health improvements, there is insufficient evidence linking specific physical housing changes to specific health outcomes, with a few important exceptions, such as lead poisoning and residential lead paint exposures (Jacobs and Nevin 2006) and multiple interventions and asthma (Morgan et al. 2004).

Despite the growing body of knowledge about health and housing, the World Health Organization (WHO) concluded that the evidence base required improvement after assessing hundreds of studies and weighing the strength of the evidence from each (WHO 2006). Of 25 housing risk factors, only 12 had “sufficient evidence” to estimate the disease burden, 11 had “some evidence,” and 2 had “insufficient evidence” (see Appendix). In addition, the report noted that there has been no trend analysis to date.

Overall life expectancy has increased in the United States between 1990 and 2004, with men and women living an average of 3.4 and 1.6 years longer, respectively [Centers for Disease Control and Prevention (CDC) 2007]. Yet certain chronic diseases have increased, including deaths from chronic lower respiratory diseases (from 28.3 deaths/100,000 in 1980 to 41.1 deaths/100,000 in 2004), doctor-diagnosed diabetes (from 8.3% in 1988–1994 to 10.2% in 2001–2004), hypertension (from 21.7% in 1988–1994 to 26.7% in 2001–2004), and overweight among children (from 4.2% in 1963–1965 to 17.5% in 2001–2004) (CDC 2007). From 1980 to 1995, 12-month asthma prevalence in children doubled, from 3.5% to 7.5%. From 2001 to 2004 asthma prevalence was constant at 7.1% (Moorman et al. 2007).

Although other risk factors are also likely to be important, the large health differences among lower-income and minority families compared with other populations suggest housing conditions may contribute to chronic disease in some populations (Rosenberg and Wilson 2001). For example, asthma rates are higher among children living in low-income communities (Mannino et al. 2002). From 2001 to 2004, asthma in children living below the federal poverty level was 10.3%, compared with 6.4–7.9% for those at or above the poverty level (Moorman et al. 2007). Inadequate housing conditions appear to be an independent contributor to the risk of diabetes in urban, middle-age African–Americans (Schootman et al. 2007). The research suggested that “the condition of an individual’s home appears to serve as a marker for some important underlying factor(s)” beyond those of diet and heredity (Grant 2007). Increases in headache and migraine could be related to neurotoxicant (e.g., pesticide) exposure in housing, which may be more common among families living in poor-quality housing that is more likely to have pest infestation problems (Julien et al. 2008).

Adult obesity rates have increased substantially, from 13.3% in 1960 to 33.9% in 2003–2004, and the rate of overweight school-age children increased from 4.0% in 1971–1974 to 18.8% in 2003–2004 (CDC 2007). Recent attention among planners and public health researchers to the built environment and health (Galster 2008; Malizia 2006) has linked housing to obesity by focusing on opportunities (or lack thereof) to exercise, walk more, and drive less to school, shopping, and/or work. A growing but disparately designed body of empirical research links obesity with location of residence, resources, television use, walkability, land use, transportation options, sprawl, and level of deprivation (Booth et al. 2005).

In addition to external conditions, obesity may also be linked to internal housing climate. A common assumption is that staying indoors contributes to obesity, especially among youth. Families living in neighborhoods with high crime rates may feel that the indoors is safest (Saelens et al. 2003). However, the increased use of central air conditioning and heating may also be contributing to rising obesity levels, because the body expends less energy in temperature ranges associated with climate-controlled settings (Keith et al. 2006). Of course, other factors are also likely to contribute to this problem.

The most advanced research to date that demonstrates the relationship between housing conditions and health is on lead poisoning. The prevalence of childhood blood lead levels ≥ 10 μg/dL has declined by 68% from 1991–1994 to 1999–2002, primarily due to changes in the nation’s older housing stock (CDC 2005; Jacobs and Nevin 2006). Multifactorial asthma interventions that rely in part on changes to housing factors have also been shown to be effective (Morgan et al. 2004). But for many other housing and health relationships, the evidence base remains wanting and requires further study (Krieger and Higgins 2002; Matte and Jacobs 2000; WHO 2006). For example, removal of carpets is often a measure employed to improve the health status of children with asthma, yet the evidence is conflicting (Custovic and Wijk 2005). Although central air conditioning and better insulation and windows to control indoor climate have the potential to reduce some health problems, including asthma and respiratory symptoms (Howden-Chapman et al. 2007; Leech et al. 2004), they can also potentially exacerbate other threats to health (e.g., carbon monoxide from a gas stove or furnace may rise in a well-sealed home if sufficient fresh air is not delivered to the living space and exhaust ventilation is deficient).

Our goal was to combine historical data from two nationally representative surveys, the American Housing Survey (AHS) and the National Health and Nutrition Examination Survey (NHANES), to identify housing trends from approximately 1970 to 2000 that could be related to changes in population health, especially children and other at-risk subpopulations. The United States does not currently have a single representative national survey that integrates both population health and housing quality, making this the first attempt to integrate retrospectively both housing and health on a nationally representative, decades-long scale.

## Methods

AHS data permit analysis of a wide array of housing and neighborhood quality, including detailed observations of interior and exterior conditions that can affect health and safety (e.g., window and floor conditions, lighting in public areas, trash), proximity to noxious and other nonhousing land uses (e.g., industrial, commercial), potential stressors (e.g., crime, noise, odors), and indicators of community investment (e.g., nearby building conditions). NHANES data provide detailed medical and biomonitoring evidence of changes in health indicators among adults and children. Both data sets contain information on socioeconomic status as well as race and ethnicity, which can be used to investigate relationships between housing and health disparities among lower-income and minority populations in the United States. Although AHS and NHANES are representative of the nation’s housing and health, respectively, neither collect data from the same individual housing units and household occupants.

We obtained national data on physical and mental health from 1971–1975 (NHANES I), 1976–1980 (NHANES II), 1988 through 30 June 1991 (NHANES III, phase 1), 1 July 1991 through 1994 (NHANES III, phase 2), and 2001–2002. The NHANES data include selected categorical and continuous health variables related to trends in housing, as well as demographic data that could modify or other- wise affect the trends identified. All statistics reported for adults 20–74 years of age are age-adjusted to the 2000 population to account for population changes (CDC 2002).

We also acquired national data from the AHS from the following years: 1973, 1979, 1989, 1993, 1997, and 2001. We selected these years because they are similar to those covered by NHANES. The AHS data include categorical and continuous variables related to trends in population health, as well as demographic data that could modify or otherwise affect the trends identified.

We first eliminated both AHS and NHANES variables that either had insufficient data over time or were not likely to be related to health outcomes or housing conditions. We then identified AHS variables that showed change over time and which plausibly could be related to health (Table 1). Similarly, we identified NHANES variables that had sufficient data across the waves of surveys to examine long-term trends that showed change (Table 2). Six variables (cough, wheeze, bronchitis, emphysema, emphysema/bronchitis, and cadmium level) had no apparent change with time, so they were not considered for the comparative analysis with the housing factors. We excluded three health variables (back pain, “ever had asthma,” and doctor-diagnosed hypertension) from further analysis because these conditions were better measured by other variables [general self-reported health status, self-report that a doctor told the subject he or she currently has asthma, and hypertension indicated by measured blood pressure with the criteria used by Chobanian et al. (2003), respectively]. In this article, we define asthma trends as the percentage of respondents who stated that a doctor told them they had asthma. NHANES did not collect consistent data on mental health status, such as depression, anxiety, and others, so these were excluded. This left seven health outcomes in NHANES that could be related to housing and neighborhood quality and for which data are available between approximately 1971 and 2002 (Table 2).

We analyzed data using SAS System for Windows, version 9.1.3 (SAS Institute Inc., Cary, NC). The chi-square test and analysis of variance were used to test the hypothesis that there was no change in a dichotomous or continuous variable across time, respectively. The tests adjusted for the unequal survey weights within AHS and NHANES as well as the clusters in NHANES.

AHS typically collects data from about 55,000 households every 2 years, and NHANES typically collects data from about 10,000 individuals, depending on the year involved. We characterized the magnitude of the relative change (not only its statistical significance) between one time period and another. We qualitatively describe a change of ±5% or more as “large,” a change of ±2–5% as “small,” a change of ±1–2% as “very slight,” and a change of less than ±1% as “no change.” We dropped the latter from further analysis. A few of the variables had both increases and decreases over various time spans. We also examined changes in survey design and administration (e.g., a change from direct observation by a survey worker to a self-report from the occupant) to discern whether methodologic factors could explain the changes observed.

## Results

Because both AHS and NHANES are large data sets, changes in all the health and housing variables that were ±1% or more were statistically significant (*p* < 0.05). Thirty-two housing variables had either small or large changes in the trend line along with possible associated health outcomes (Table 1). The largest increases were in neighborhood crime (crime went up and then down), central air conditioning, and single-family homes nearby, and the largest improvement was in fuses blown/circuit breakers tripped, water leakage from outside, neighborhood public transportation, bad smells in the neighborhood, and traffic. None of the housing variables showed large declines in housing quality between 1970 and 2000.

NHANES data show that, with the exception of childhood asthma (which saw a large increase), the size of the trends among adults and youth were in the same categories across numerous health variables (Table 2). The largest change in health outcomes for adults over time was improved general health status, although there were large increases in body mass index (BMI) and diagnosed diabetes at the same time. There were large decreases in blood lead concentration, serum cotinine concentration, and hypertension. For youth, the trend for overall general health was a large increase after a large decrease. During this time, however, whereas lead poisoning and cotinine largely declined, asthma and BMI greatly increased.

**Figure 1:**
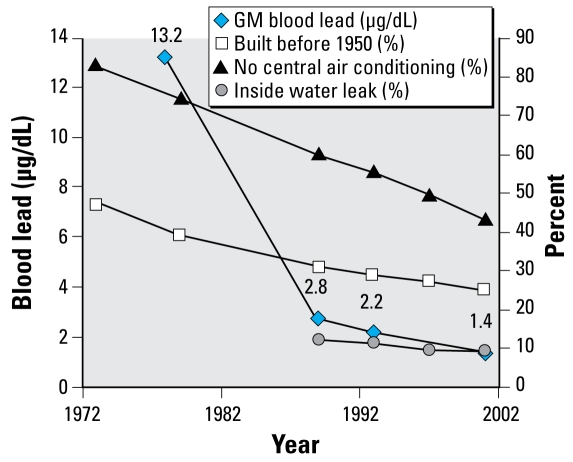
Changes in children’s blood lead (μg/dL), housing age, leaks, and central air conditioning over time. Abbreviation: GM, geometric mean.

**Figure 2:**
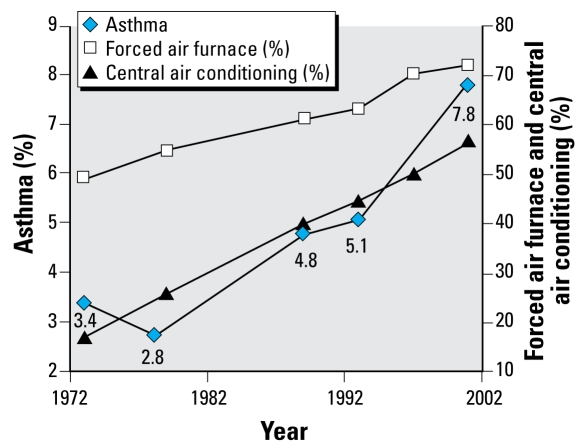
Changes in asthma, central air conditioning, and forced air furnaces over time.

**Figure 3:**
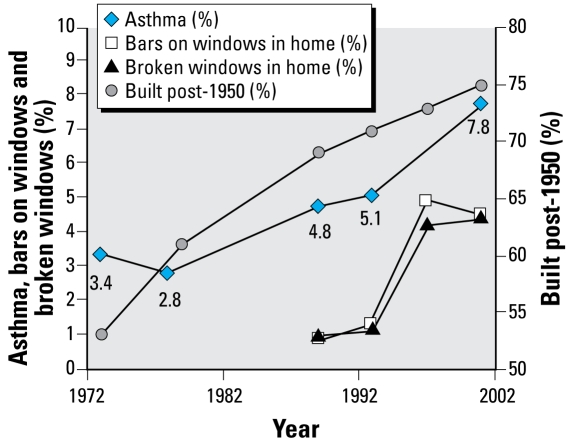
Changes in asthma, bars on windows, broken windows, and year built over time.

**Figure 4:**
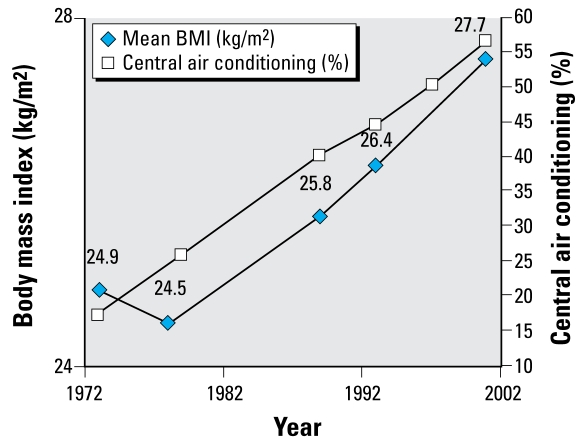
Changes in BMI (kg/m^2^) and central air conditioning over time.

**Figure 5:**
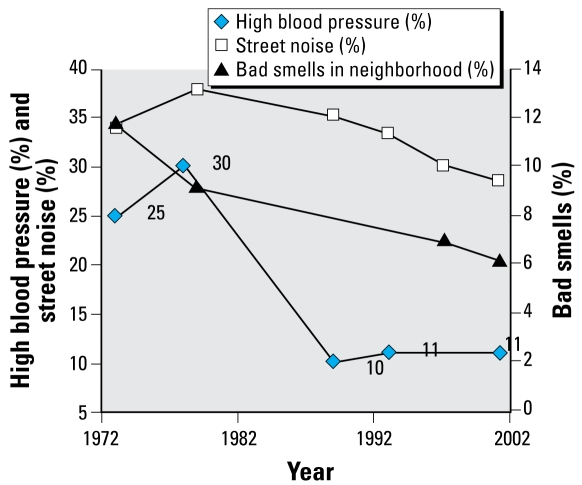
Changes in high blood pressure, street noise, and bad smells in neighborhoods over time.

Housing and neighborhood variables in the AHS over the same time period were placed in one of the six health categories we used for NHANES and then analyzed by magnitude of change (Table 3). We plotted specific housing and health variables over time (Figures 1–5). When cross-tabulated this way, overall general health saw little change compared with changes in housing conditions, so no further analysis was performed for the general self-reported health variable. But specific changes in housing and neighborhood condition did follow more specific health outcome variables, including blood lead (Figure 1), asthma (Figures 2 and 3), BMI (Figure 4), and cardiovascular health (high blood pressure; Figure 5). Instances in which housing and health trends did not follow each other are described elsewhere (National Center for Healthy Housing 2008). On the figures, we plot the central year of the particular multiyear NHANES wave.

**Figure 6:**
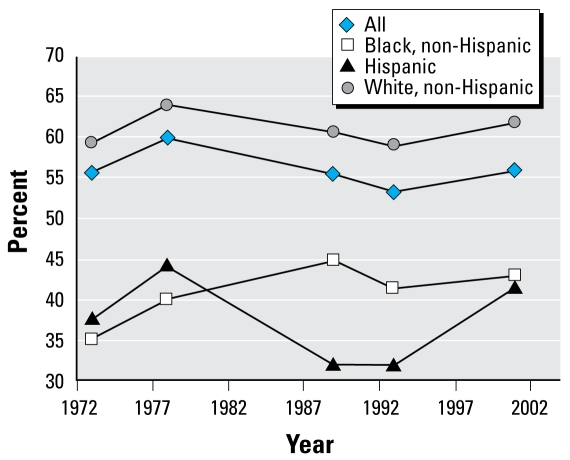
Excellent or very good general health status by race/ethnicity.

We also examined the trends in health and housing outcomes for different racial/ethnic subpopulations. For example, we included “excellent” or “very good” general health status by race/ethnicity (Figure 6). Our analysis supported previous findings that some racial groups live in poorer-quality housing [U.S. Department of Housing and Urban Development (HUD) 2003] and have poorer health than the general population (Kawachi et al. 2005). We found little or no change in these disparities over time.

## Discussion

The results support five trends: *a*) housing age (quality) and amenities trend with lead poisoning over time; *b*) changes in heating and air conditioning systems and prevalence of broken windows and bars on windows trend with the prevalence of asthma; *c*) housing air conditioning trends with obesity; *d*) cardiovascular health trends with changes in proximity to open space, commercial and industrial facilities, noise, and neighborhood air quality; and *e*) general health status by race/ethnicity has remained much the same and follows trends in housing over time.

### Housing age (quality) and amenities trend with lead poisoning over time

The association between lead poisoning and housing condition is perhaps the most robust, given the large body of evidence showing that exposure to residential lead-based paint hazards causes increased blood lead levels (HUD 2000; Jacobs 1995; Levin et al. 2008). From 1976 to 2002, NHANES data show that blood lead levels declined from 13.2 μg/dL to 1.4 μg/dL in children younger than 13 years of age. We examined children < 13 years of age because other NHANES variables were collected only for this subset of the population. The trends are the same for children younger than 6 years. Of course, over this time period, reductions in other nonhousing sources of lead also occurred, such as gasoline, food canning, and industrial emissions. It is noteworthy that the slope in blood lead is much steeper between 1978 and 1988, suggesting that the phaseout of multiple sources (especially lead in gasoline) was effective and that the slower rate of decline in the following years reflects the slower changes in housing lead-based paint hazards (Jacobs et al. 2002).

The decrease in blood lead level from 1990 to 2000 is associated with trends in housing demolition and substantial rehabilitation, including lead paint abatement (Jacobs and Nevin 2006). Both demolition and housing rehabilitation are associated with trends in housing age, because older housing units are more likely to be demolished and rehabilitated. As the number of older housing units declines (as reported in AHS), the prevalence of lead-based paint can also be expected to decline, because older housing is more likely to have deteriorated paint and that paint is more likely to be lead-based paint. The disparity in lead poisoning by race and ethnicity has also been reduced dramatically (CDC 2005), suggesting that housing improvements can result in reduced health disparities in disadvantaged populations.

Those housing conditions that affect interior moisture can also be expected to affect paint film condition, because lower moisture levels improve paint film durability (Figure 1). In short, the reduced water leakage from both the interior and the exterior that was reported in AHS can be expected to positively affect paint quality, which in turn could be linked to the reduction in both child and adult blood lead levels seen in NHANES. Similarly, the large decrease in homes without central air conditioning can also be expected to decrease interior moisture levels, thus increasing paint film durability and reducing exposures from lead-based paint hazards. For older homes built before 1978 and before 1950, interior and exterior leaks were more likely than in newer homes (*p* < 0.001), consistent with earlier findings that such houses have more lead hazards (Jacobs et al. 2002). The older homes were also less likely to have central air conditioning (*p* < 0.001; data not shown).

### Changes in heating and air conditioning systems and prevalence of broken windows and bars on windows trend with the prevalence of asthma

Those housing factors that follow the large historic increase in asthma include the large increase in forced air furnaces and central air conditioning (Figures 2 and 3). Even though such furnaces are typically equipped with filtration systems, they can be expected to result in higher airborne particulate matter due to higher air velocities that cause resuspension of dust particles that otherwise would settle out of the air. Furthermore, in single-family housing, such systems typically do not introduce fresh air, relying instead on building leakage for fresh air supply. Reduced fresh air introduction can be expected to increase exposure to allergens, oxides of nitrogen, and other airborne asthma triggers because they will not be as diluted.

The increase in central air conditioning discussed above may also contribute to asthma, because such systems will reduce fresh air infiltration, even as they improve thermal comfort. Reduced fresh air infiltration may lead to increased exposure to oxides of nitrogen and other asthma triggers and by- products of combustion from cooking stoves and other sources. Windows that would otherwise be open in the warm months are more likely to be kept closed in the presence of central air conditioning. Central air conditioning will also increase air velocities and resuspension of particulate matter. On the other hand, pollen infiltration and other outdoor asthma triggers could be reduced, and lower indoor humidity could reduce dust mite prevalence, all of which could have beneficial effects. Why the decrease in pre-1950 housing units is associated with an increase in asthma in this study is unknown, although one possible explanation is that construction materials in newer housing tend to rely more on man-made materials. For example, certain types of new flooring may contain phthalates, which have been implicated as an asthma trigger (Jaakkola and Knight 2008). Unfortunately, AHS does not track the use of such materials.

Finally, broken windows and bars on windows are both measures of stress, which is also thought to be related to asthma (Figures 2 and 3).

### Housing air conditioning trends with obesity

The increase in central air conditioning and its associated improved thermal comfort could provide an incentive for people to remain indoors and thus exercise less and/or to exert less energy through lower metabolic rates (Figure 4).

### Cardiovascular health trends with changes in proximity to open space, commercial and industrial facilities, noise, and neighborhood air quality

NHANES assessed hypertension in several different ways, including measured high blood pressure (Chobanian et al. 2003) and respondent report of physician–diagnosed hypertension. The presence of commercial buildings in residential areas could be associated with increased walking to nearby services, including medical care. This might also be attributed to improvements in outside conditions in general, but we did not observe such effects here (data not shown). However, Figure 5 shows an increase in high blood pressure in the late 1970s and an increase in bothersome street noise, followed by a decline in both. The trend in “bad smells” shows a long-term decrease. Both may be attributed to increased use of zoning, which aims to separate noxious uses and high traffic from residential areas, as well as the decrease in industrial manufacturing in urban areas. More land is zoned now due to increased development.

### Trends in general health status by race/ ethnicity have remained much the same and follow trends in housing over time

Figure 6 shows that health disparities have changed little over the past 30 years. NHANES has collected information on self-reported general health status using a 5-point scale from 1973 to 2002 (excellent, very good, good, fair, or poor). The use of self-reported general health status has been found to be a good predictor of health (Miilunpalo et al. 1997). Those adults between 20–74 years of age whose self-reported general health status was either “excellent” or “very good” increased from 47.8% in 1973 to 54.8% in 2001. When corrected for age, the improvement was far smaller (55.6% and 55.9% in 1973 and 2001, respectively), with larger variations in the intervening years. For adults 40–60 years of age, excellent or very good general health increased from 43.4% in 1971 to 53.6% in 2002. Among those between 13 and 20 years of age, there was a large decrease in “excellent” or “very good” health, from 69.5% in 1975 to 50.5% in 1991, but then a large increase to 69.2% in 2002. Although the increase in life span is often associated with improved population health, it is possible that the overall trend in general health differs across age groups, which makes it difficult to show how general health status changes follow housing quality. Theoretically, the following housing trends could be linked to general health: An increase in dwelling unit size could be associated with reduced crowding. Newer housing could be associated with reduced potential for injuries. Reduced proximity to industrial pollution through zoning could reduce exposures to airborne contaminants. A decrease in basements could be associated with reductions in infiltration of soil gases and moisture. A decline in the use of private drinking water supplies could be associated with reductions in waterborne diseases. Finally, declines in prevalence of pests could be associated with reductions in communicable diseases and reduced exposure to pesticides. However, using these data sets, we were not able to demonstrate how any of these housing factors followed trends in general health, because it is likely that many factors beyond housing quality contribute to general health status (Zahran et al. 2005). More precise definitions of general health status and the associated environmental conditions that affect it are clearly needed to help identify those housing conditions related to general health for both the entire population and disadvantaged populations.

### Limitations

In 1997, the AHS implemented a major revision to its survey instrument and methodology. For example, ovens and refrigerators not only had to be present to have a complete kitchen (as required pre-1997), but they also had to work. Questions about neighborhood quality in the survey instrument were reordered, which may have influenced the general increase in satisfaction with the neighborhood. AHS began using a respondent questionnaire to capture most exterior and neighborhood data, instead of direct observation by a data collector. As a result, not only did the person assessing the conditions change, but more homes were surveyed and the proportion of single-family homes increased. These changes could have introduced a bias in the direction of improved housing and neighborhood quality, although its magnitude remains uncertain. Because these methodological changes could explain some (or all) of the temporal variation seen in the data for 1997 and later years, we have noted them in Table 3.

The methods used in NHANES also changed over the time period examined here, which could influence the findings. In 1999, NHANES began a continuous data collection process as opposed to episodic data collection in earlier years. Survey administration and data collection variables both contribute to the possibility that changes from one NHANES survey to the next could be due to methodologic issues instead of actual changes. For example, the early NHANES provided less precision on some ethnic minority groups, and infants and the elderly were not included, whereas in later years these groups were included (CDC 2008). This could cause some population health metrics to decrease because these subpopulations are generally in poorer health. Categorical measures of certain health outcomes also changed over the years. For example, methods of measuring blood pressure changed (Qureshi et al. 2005), which could have limited the ability to identify true changes in this important health outcome. In addition, NHANES does not include those who were institutionalized, did not have telephones, or were homeless, all of whom can be expected to have poorer health status (Zahran et al. 2005). Finally, NHANES does not measure certain socioeconomic variables, such as wealth and type of employment, and each NHANES survey is cross-sectional, which limits the ability to control for recent onset of chronic illness or injury.

This study examined only two data sets, but there are many others that could not be examined here due to funding limitations. These other data sets include, for example, the Residential Energy Consumption Survey, the National Health Interview Survey, the U.S. Census, the American Community Survey, and the American Healthy Homes Survey. Another limitation is that rigorous statistical tests of associations between housing and health could not be conducted, because although each survey is nationally representative, the data are for different homes and individuals. It would be far superior to measure such relationships in a survey that combined housing, health, and community data in an integrated fashion. One model for this is the LARES (Large Analysis and Review of European Housing and Health Status) study in Europe (Bonnefoy et al. 2003).

Because a change in a given housing characteristic does not necessarily result in an instantaneous change in health status, differing “lag periods” might be important to determine the best fit in the two trends. A lag period is defined as the time period beginning from when an observed change in a housing variable has been identified and ending when a corresponding change in a health variable has been found. Lag periods based on best fit to the data should be estimated in future work, but more years of data would be needed.

The associations presented here are not necessarily causal. The ecologic study design makes some of the associations speculative and could be biased. Other competing explanations for changes in health status cannot be controlled through ecologic study designs such as this. For example, changes in obesity are likely related to many other factors, such as availability of fast food, decreased opportunity for exercise, increased TV watching, and changes in fat content of food. Similarly, changes in general health status could be due to factors other than those identified here, such as education, greater health awareness, lifestyle changes, economic cycles, housing affordability, health insurance status, marital status, aging population, tendency of youth to engage in risky behaviors, occupation, and chronic illnesses (Zahran et al. 2005). Nevertheless, ecologic study designs are helpful in identifying the extent of a public health problem, possible causal associations, and the need for analytic epidemiologic investigation (Merril and Timmreck 2006). Although this study cannot quantify the extent to which a given housing or neighborhood factor affects health status, it does identify avenues for more focused and refined studies. Future studies should use some of the other data sets identified above to validate these findings and to quantify any bias associated with NHANES or AHS. Although controlled trials can randomize interventions and perhaps identify specific causes and effects, they are of limited size and duration, the recruitment of control groups poses special ethical issues (particularly in housing intervention studies), and they are unlikely to be representative of the general population due to study effects. In short, the study design used here has the unique advantage of a much larger data set, covering many more housing and health variables and subjects over a much longer time period.

## Conclusions

Until recently, housing research in the United States has been concerned primarily with rising housing costs and affordability, and medical research has been concerned primarily with treatment of existing disease, not prevention through analysis of social determinants of health. But one British study found that health care costs for the occupants were reduced by 7-fold when their low-income housing was substantially rehabilitated (Ambrose 2001). An informed discussion on the popular subject of the high costs of medical insurance should include social determinants of health, including housing quality. This can also include rethinking how we account for the costs of inadequate housing and poorly designed communities, which are still largely hidden and not reflected in market pricing and program priorities. Instead, these costs are transferred to the already overburdened medical care system in the form of preventable disease and injury (Jacobs 2004). Similarly, an informed discussion of the high cost of medical insurance needs to address the effect of increasing housing cost burdens, because more income must be devoted to treating housing related preventable disease and injury. Although housing and health officials worked closely together more than a century ago to establish housing laws that helped to protect the public from diseases such as tuberculosis (Stein 1950), the need to reestablish such collaboration at local, state, and regional levels has only recently been addressed by the U.S. Surgeon General (National Institutes of Health 2005) and by HUD (1999).

Housing interventions improve health (NCCH and CDC 2008). The analysis we present here examined housing and health relationships that have been previously explored in depth, such as the effect of housing on lead poisoning, and other potential relationships that have received relatively little attention, such as housing and obesity/diabetes. The study design used here is a useful way to explore many other potential housing and health relationships, such as whether the increasing size of housing, growth in central air conditioning, increased forced air ventilation, and other factors may be contributing to obesity, diabetes, and asthma. Finally, the failure to show improvements in housing and health disparities in low-income and minority racial and ethnic groups over the substantial time horizon analyzed here is a sobering reminder that more targeted and more effective approaches are still needed.

## Figures and Tables

**Table 1:** Selected AHS trends and association with health.

Variable	Trend	Possible health associations
Crime in neighborhood		
Neighborhood has crime	Large increase in 1970s, peak 1989–1993, then slightly lower	Obesity and diabetes (stay indoors), respiratory health (stress), general health
Crime is bothersome		
Crime is so bad want to move		

Heating/cooling		
Central air conditioning	Large increase	Asthma (reduced mites), lead (reduced paint deterioration from low humidity), respiratory health, obesity and diabetes, general health
Room air conditioners	Slight decrease	

Housing characteristics		
Basement (if single family)	Slight decrease	Respiratory, general health
Unit has usable fireplace	Slight increase	Respiratory, general, cardiovascular health
Garage (for 1973 only included owner-occupied units)	Slight decrease (excluding 1973)	Respiratory, general health, cardiovascular due to carbon monoxide
Peeling paint or plaster inside	Slight decrease	Lead poisoning, respiratory health
Fuses blown/circuit breakers tripped	Large decrease	General health
Flush toilets not working at any time in last 3 months	Slight decrease^a^	General health
Common hallway lights work^b^	Small decrease	General health
Concealed wiring	Slight increase	General health
Every room has working electrical plug	Slight increase	General health
Complete plumbing facilities	Slight increase	Respiratory, general health
Firmly attached common area stair railings^b^	Slight increase in 1970s to early 1990s, then slight decrease	General health
Mice or rats seen recently	Slight decrease	Respiratory, general health
Inside water leaks in last 12 months	Slight decrease	Lead poisoning, respiratory health
Water leakage from outside	Large decrease	Lead poisoning, respiratory health

Neighborhood		
Abandoned/boarded up/vandalized buildings within half block^b^	Slight decrease	Respiratory, general health
Buildings with bars within half block^b^	Slight increase in late 1980s, followed by slight decrease	Respiratory, general health
Commercial/institutional/industrial buildings within 300 ft^b^	Slight increase in 1970s, decrease in 1980s, then large increase in 1990s	Respiratory, general, cardiovascular health
Open spaces within half block^b^	Slight increase in the early 1990s, slight decrease in late 1990s	Obesity and diabetes, general, cardiovascular, respiratory health
Single-family or apartment buildings < 4 stories within 300 ft^b^	Large increase in early 1990s, followed by flat trend	Respiratory, general, cardiovascular health
Apartment buildings 4–6 stories within half block^b^	Slight increase followed by flat trend	General health
Satisfactory public transportation^c^	Large increase since early 1970s, followed by slight increase	Obesity and diabetes, respiratory, cardiovascular, general health

Noise		
Neighborhood has heavy street noise/traffic^c^	Large decrease	Respiratory, general health
Neighborhood street noise/traffic bothersome^c^	Large decrease	Respiratory, general health
Street noise/traffic so bad you want to move^c^	Slight decrease in early 1970s, followed by other slight decreases	Respiratory, general health

Odors		
Neighborhood has bad smells^c^	Large decrease	Respiratory, general health
Neighborhood smells are bothersome^c^	Slight decrease	

Added/replaced insulation	Slight increase in early 1980s, slight decrease in 1990s, then slight increase	Respiratory, general, cardiovascular health

a Possible AHS error that increased positive responses.

b Change in 1997 to respondent report, not observation.

c Question wording and order changed in 1997.

**Table 2:** Selected NHANES health outcomes, 1971–2002.

Category	NHANES variable	Adult trend	Child trend
General	Excellent or very good health (compared with good, fair, or poor)	Large increase	Large decrease to 1988–1991, followed by large increase
Lead	Blood lead concentration (μg/dL)	Large decrease	Large decrease
Respiratory	Currently have asthma (yes/no)	Small increase	Large increase
	Serum cotinine concentration (ng/mL)	Large decrease	Large decrease
Obesity	BMI	Large increase	Large increase
Diabetes	Diagnosed with diabetes (yes/no)	Large increase	No change
Cardiovascular	Hypertension (as measured by blood pressure)	Large decrease	Not applicable

**Table 3:** Health and housing trends by size of change, 1975 to 2000.

	AHS variable			
NHANES variable	Large increase	Large decrease	Small increase	Small decrease
General health	Residence in urban area	Fuses blown	Fireplace	Basement
	Central air	Neighborhood street noise^b^	Bars on windows^a^	Garage
	Public transport satisfactory^b^	Neighborhood has bad smells^b^	Plug in every room	Flush toilets not working
	No. of bedrooms, size	Number of older units	Complete plumbing	Common area lights working^a^
	Cost burden	Minor junk accumulation	Commercial buildings in residential areas^a^	Firmly attached railings^a^
		Outside water leaks	Open spaces within half of a block^a^	Rats or mice seen
			Home ownership	No. of rental units
			No. of adequate housing units	Inside water leaks
				Abandoned buildings within half block^a^
				Private wells
				No. of severely inadequate units

Asthma	Neighborhood has crime		Fireplace	Common area lights working^a^
	Central air		Bars on windows^a^	Mice or rats seen
	Warm (forced air) furnaces		Broken windows^a^	

Obesity	Neighborhood has crime		Open spaces within half of a block^a^	
	Central air			
	Public transportation satisfactory^b^			
	No. of bedrooms, size			
	Cost burden			
	Residence in urban area			

Diabetes	Central air			
	No. of bedrooms			
	Cost burden			
	Residence in urban area			

Lead	Central air	Percent older units		Peeling paint, holes in floors

Cotinine	No. of bedrooms, square feet (size)	Neighborhood has bad smells^b^	Open spaces within half block^a^	

Cardiovascular			Fireplace	
			Commercial buildings in residential areas^a^	
			Open spaces within half of a block^a^	

a Question changed from observer to survey respondent response in 1997.

b Question wording and order changed in 1997.

## Appendix 1. WHO Assessment of Evidence Linking Health and Housing

### Linkages with sufficient evidence for estimating burden of disease

#### Physical factors

- Heat and related cardiovascular effects and/or excess mortality
- Cold indoor temperatures and winter excess mortality
- Energy efficiency of housing and health
- Radon exposure in dwellings and cancer
- Neighborhood and building noise and related health effects

#### Chemical factors

- Environmental tobacco smoke exposure in dwellings and respiratory and allergic effects
- Lead-related health effects

#### Biological factors

- Humidity and mold in dwellings and related health effects
- Hygrothermal conditions and house dust mite exposure

#### Building factors

- Building and equipment factors and injuries/domestic accidents
- Injury database on domestic accidents and injuries
- Estimating the number of home accidents from injuries

#### Social factors

- Multifamily housing, high-rise housing, and housing quality and mental health

### Linkages with some evidence for estimating burden of disease

#### Physical factors

- Ventilation of the dwelling and respiratory and allergic effects
- Chemical factors
- Volatile organic compounds and respiratory, cardiovascular, and allergic effects

#### Biological factors

- Cockroaches and rodents in dwellings and respiratory and allergic effects
- Pets and mites and respiratory, allergic, or asthmatic effects

#### Building factors

- Sanitation and hygiene conditions and related physical health effects

#### Social factors

- Social conditions of housing and fear/fear of crime
- Poverty and social exclusion and related health effects
- Crowding and related health effects
- Social factors/social climate and mental health

### Linkages with insufficient evidence for estimating burden of disease

#### Physical factors

- Lighting conditions in the dwelling and mental and other health effects
- Particulate matter in indoor air and respiratory and allergic effects
